# Difference in Parent-Child Care Experience by Demographic Characteristics: Focusing on Stress, Needs, and Meaning

**DOI:** 10.1093/geroni/igaa057.3338

**Published:** 2020-12-16

**Authors:** Ayako Baba

**Affiliations:** Kanazawa University, Tokyo, Japan

## Abstract

OBJECTIVE: In super aging society, unpaid caregivers play important roles. Therefore, they should be supported to realize the lives they want. However, it is unclear what caregivers’ particular needs are under different conditions. This study aimed to explore the difference in forms of stress and needs of caregivers by demographic characteristics. METHOD: Adult children who were caring for or had cared for their parents at home under the Japanese long-term care insurance system (n=653) completed the three open-ended questions concerning caregiving stress, unmet needs, and meaning, combined with a demographic survey. Data were analyzed using text mining approaches such as correspondence analysis and co-occurrence network analysis, which illustrated differences in description according to demographic variables. RESULT: 1) Caregivers of fathers felt stressed when rebuked by care-recipients, whereas daughter caregivers of mothers felt stressed in coping with care-recipient dementia and complaint, and balancing caregiving with parenting. 2) Caregivers in economic difficulty needed instrumental and financial support, whereas others needed flexible support and accessible consultation services. 3) Caregivers living with care-recipients found meaning in experience and repaying parents for raising them, whereas caregivers living away found meaning in improved parent-child relationships and images of care-recipients. Caregivers of persons with dementia found meanings in sharing time of tranquility with care-recipients. CONCLUSION: The study revealed differences in caregiving experience by care dyads, economic situation, living arrangement, and dementia etc. These results suggest that family caregivers should be provided with services to help find meanings in work and reduce stress levels, according to caregiving conditions.

